# Relación entre sobrecarga del cuidador y agencia de autocuidado del paciente de cirugía cardiaca en Cúcuta - Colombia*

**DOI:** 10.15649/cuidarte.2433

**Published:** 2022-10-22

**Authors:** Débora Milena Álvarez-Yáñez, Claudia Ximena Reyes-González

**Affiliations:** 1 . Universidad Francisco de Paula Santander, Cúcuta. Colombia. Email deboramilenaay@ufps.edu.co Universidad Francisco de Paula Santander Colombia; 2 . Universidad Francisco de Paula Santander, Cúcuta. Colombia. Email claudiaximenarg@ufps.edu.co Universidad Francisco de Paula Santander Universidad Francisco de Paula Santander Cúcuta Colombia claudiaximenarg@ufps.edu.co

**Keywords:** Periodo Posoperatorio, Cirugía Torácica, Cuidadores, Salud de la Familia, Atención de Enfermería, : Postoperative Period, Thoracic Surgery, Caregivers, Family Health, Nursing Care, Período Pós-Operatório, Cirurgia Torácica, Cuidadores, Saúde da Família, Cuidados de Enfermagem

## Abstract

**Introducción:**

Durante el posoperatorio de cirugía cardiaca surgen problemas físicos, psicológicos y sociales, que afectan la capacidad de agencia de autocuidado del paciente, lo cual trae consigo la necesidad de un cuidador durante su recuperación.

**Objetivo:**

Determinar la relación entre el nivel de sobrecarga del cuidador principal y la agencia de autocuidado del paciente de cirugía cardiaca.

**Materiales y Métodos:**

Se planteó un diseño descriptivo de corte transversal, analítico correlacional, participaron pacientes (n=86) y cuidadores (n=86) del programa de cirugía cardiaca, en una institución de alta complejidad en Cúcuta - Colombia, seleccionados con muestreo no probabilístico por cuotas, se aplicó la escala de valoración de agencia de autocuidado ASA y la escala de sobrecarga del cuidador de Zarit.

**Resultados:**

En cuidadores con una sobrecarga intensa, el 83,33% tenían a su cargo pacientes con baja capacidad de autocuidado. En el grupo de cuidadores con sobrecarga leve, el 63% los pacientes tenían capacidad de agencia de autocuidado media, mientras que, los cuidadores sin sobrecarga, la mayoría de sus pacientes (71, 01%) tenía una capacidad de agencia de autocuidado media, pero un 20,29% exhibió una alta capacidad de autocuidado.

**Discusión:**

La capacidad de agencia de autocuidado reduce el nivel de sobrecarga del cuidador, cuando el paciente logra adaptarse a las actividades que contribuyen al cuidado, disminuye el nivel de dependencia.

**Conclusiones:**

La investigación demostró la relación entre estas dos variables, se debe abordar a pacientes y cuidadores para alcanzar el empoderamiento del autocuidado.

## Abstract (en)

**Introduction:**

During cardiac surgery postoperative care, physical, psychological, and social problems arise, affecting the patient's self-care agency, which brings with it the need for a caregiver during recovery.

**Objective:**

To determine the relationship between primary caregivers' burden level and the self-care agency of cardiac surgical patients.

**Materials and Methods:**

A descriptive, cross-sectional, analytical, and correlational design was used. Patients (n=86) and caregivers (n=86) attending a cardiac surgery program from a high complexity medical center in Cúcuta, Colombia, participated and were selected using non-probability quota sampling. Self-care Agency Scale (SAS) and Zarit Burden Interview were used in the study.

**Results:**

Among the caregivers with severe burden, 83.33% cared for patients with low self-care agency. In the mild-burden caregivers' group, 63% of the patients had middle self care agency. Most patients (71.01%) of caregivers with no burden had middle self-care agency, but 20.29% exhibited high self-care agency.

**Discussion:**

the capacity for self-care agency reduces the level of caregiver burden, when the patient manages to adapt to the activities that contribute to care, the level of dependency decreases.

**Conclusion:**

The research demonstrated the relationship between these two variables. Patients and caregivers must be addressed to achieve self-care empowerment.

## Resumo

**Introdujo:**

Durante o pós-operatório de cirurgia cardíaca surgem problemas físicos, psicológicos e sociais que afetam a capacidade de agencia de autocuidado do paciente, o que traz consigo a necessidade de um cuidador durante a recuperado.

**Objetivo:**

Determinar a relagáo entre o nivel de sobrecarga do cuidador principal e a agencia de autocuidado do paciente de cirurgia cardíaca.

**Materiais e Métodos:**

Foi proposto um desenho descritivo transversal, analítico correlacional, participaram pacientes (n=86) e cuidadores (n=86) do programa de cirurgia cardíaca de uma instituido de alta complexidade em Cúcuta - Colombia, selecionados com amostragem náo probabilística por cotas, foram aplicadas a escala de classificado da agencia de autocuidado ASA e a escala de sobrecarga do cuidador de Zarit.

**Resultados:**

Nos cuidadores com sobrecarga intensa, 83,33% eram responsáveis por pacientes com baixa capacidade de autocuidado. No grupo de cuidadores com sobrecarga leve, 63% dos pacientes apresentaram média capacidade de agencia de autocuidado, enquanto, nos cuidadores sem sobrecarga, a maioria de seus pacientes (71,01%) apresentou média capacidade de agencia de autocuidado, mas 20,29% apresentaram alta capacidade de autocuidado.

**Discussao:**

A capacidade de agencia de autocuidado reduz o nível de sobrecarga do cuidador, quando o paciente consegue se adaptar as atividades que contribuem para o cuidado, o nível de dependencia diminui.

**Conclusóes:**

A pesquisa mostrou a relado entre essas duas variáveis, pacientes e cuidadores devem ser abordados para alcanzar o empoderamento do autocuidado.

## Introducción

La enfermedad coronaria es una de las principales causas de muerte y discapacidad en todo el mundo^1^. Según el informe de la Organización Mundial de la Salud (OMS) del año 2017, las enfermedades no transmisibles ocasionan 41 millones de muertes cada año, siendo el 71% de la mortalidad en el mundo por cardiopatía isquémica y accidente cerebrovascular durante los últimos 15 años^2^. Anualmente, en países desarrollados más de 2.200 personas mueren a causa de enfermedades cardiovasculares^3^.

En casos de obstrucción de las arterias coronarias que por su localización o extensión peligra la vida del paciente, es necesario realizar la revascularización quirúrgica^1^. Sin embargo, los pacientes enfrentan problemas físicos, psicológicos y sociales después de cirugía requiriendo atención en casa. Cabe resaltar que, la prestación de cuidados no acostumbra a repartirse por igual entre familiares, sino que existe un cuidador principal, en quien recae la mayor responsabilidad del cuidado^4^. Para esta investigación, la agencia de autocuidado se define como una conducta sobre sí mismo, para regular los factores que afectan su salud y promover su proceso de rehabilitación^5^. En el posoperatorio previene la recurrencia de infarto de miocardio, cirugía adicional y muerte prematura^4^.

Con respecto a la sobrecarga del cuidador, es definida como el grado afectación de la persona encargada del cuidado^6^. Siendo así, algunas investigaciones han mostrado que existen factores influyentes, como son las actitudes frente a la experiencia del cuidado y la disponibilidad de recursos del sistema familiar que incrementan el estrés en cuidadores^7^^-^^8^. Es primordial, el apoyo social que favorezca una relación de confianza entre paciente y cuidador para lograr una adecuada agencia del autocuidado. Aunque, es considerado una estrategia eficaz, no es fácil, requiere apoyo y motivación para mejorar la calidad de vida^1^^-^^9^. Es necesario el rol de enfermería con los cuidadores para adquirir esas conductas adaptativas^10^.

De acuerdo con lo argumentado, para los profesionales de enfermería es importante investigar sobre este fenómeno. Se planteó esta investigación con el objetivo determinar la relación existente entre el nivel de sobrecarga del cuidador principal y la agencia de autocuidado del paciente de cirugía, basado en el modelo teórico de Dorothea Orem. Las hipótesis de esta investigación fueron:

H1: Existe asociación entre el nivel de sobrecarga del cuidador principal y la agencia de autocuidado del paciente de cirugía cardiaca.
H2: El nivel de agencia de autocuidado de los pacientes depende de sus características sociodemográficas como la edad, el sexo, el estado civil, el nivel socioeconómico y la ocupación. H3: El nivel de agencia de autocuidado mejora con el tiempo de recuperación (posoperatorio). H4: El nivel de sobrecarga de los cuidadores depende de sus características sociodemográficas como la edad, el sexo, el estado civil, el nivel socioeconómico y la ocupación
H5: El nivel de sobrecarga del cuidador está relacionado con el tiempo que lleva desempeñando su rol.

## Materiales y métodos

Estudio descriptivo de corte transversal, analítico correlacional entre agencia de autocuidado y nivel de sobrecarga del cuidador en una institución de alta complejidad en Cúcuta - Colombia. Los pacientes que participaron en el estudio cumplieron con los criterios de pertenecer al programa de cirugía cardiaca año 2017; ser mayor de 18 años con competencias de lectura y escritura, deseo de participación. El cuidador principal debe ser mayor de 18 años, con competencias de lectoescritura, deseo de participación.

Se excluyeron personas con alteraciones mentales y/o físicas documentadas en historia clínica, con afectación cognitiva; pacientes con complicaciones en el posoperatorio que requieran hospitalización.La población estuvo conformada por 211 pacientes y su cuidador principal. El cálculo del tamaño de la muestra corresponde al contraste de hipótesis para diferencia de medias^11^, se estableció un tamaño muestral n= 137 pacientes e igual número de cuidadores, nivel de confianza 95% y margen de error 5%. Sin embargo, a medida que avanzó la investigación se presentaron limitaciones en la recolección de información, razón por la cual se utilizó un muestreo no probabilístico por cuotas, se tomaron todos los pacientes del programa de cirugía cardiaca de una institución de alta complejidad en Cúcuta - Colombia, operados en el año 2017 y su cuidador principal, que cumplieron con los criterios de inclusión. El análisis final se realizó con 86 pacientes y 86 cuidadores, la perdida de los sujetos estuvo alrededor del 59%, específicamente por, mortalidad del paciente 16% (34 pacientes), sin contactar 25 % (52 pacientes) y 18% no aceptaron participar (39 pacientes)^12^.

Para la medición de las variables del estudio se aplicaron dos instrumentos:

Escala de valoración de agencia de autocuidado (ASA) desarrollada por Isenberg y Evers, para medir el concepto central de la Teoría de Enfermería de Déficit de autocuidado de Orem, se utilizó la versión en español modificada en el año 2004 en Colombia, por la profesora de la facultad de Enfermería de la Universidad Nacional de Colombia, Edilma Reales, con un alfa de Cronbach 0,68 y corregido 0,74. Está compuesta por 24 ítems, respuesta tipo Likert con 4 apreciaciones, distribuidas en: Nunca 1, casi nunca 2, casi siempre 3 y siempre 4, permitiendo una posible puntuación entre 24 y 96 puntos, los ítems 6,11 y 20 son negativos; se considera una agencia de autocuidado baja (24 -48 puntos), regular (49-72 puntos) y buena (73-96 puntos)^13^.

Escala de sobrecarga del cuidador de Zarit (ZCBI), diseñada por Zarit, Rever y Bach-Peterson en 1983, consistencia interna de 0.91 y fiabilidad test-retest de 0.86. Está compuesta por 22 ítems con respuesta tipo Likert de 5 puntos (0 = Nunca; 1 = Casi nunca; 2 = A veces; 3 = Bastantes veces; 4 = Casi siempre), se puede obtener una puntuación global que oscila entre 0 y 88 puntos^14^.

La recolección de la información se llevó a cabo en el periodo de enero a diciembre año 2017, a través de entrevista presencial con el paciente y su cuidador, se contemplaron los aspectos éticos universales de investigación con seres humanos^15^. Se mantuvo la confidencialidad y privacidad de los participantes, su autonomía para decidir su participación y retiro voluntario, se dejó copia del consentimiento informado escrito por parte de los pacientes y su cuidador previo a la administración de los instrumentos. Esta investigación conto con el aval del autor de la escala, así como la aprobación del Comité de ética de la institución participante, acta 031- 01/12/2016.

Además, se tuvo en cuenta lo dispuesto en la Resolución 008430 de 1993 por la cual se establecen las normas científicas, técnicas y administrativas para la investigación en salud. El análisis estadístico se basó en la elaboración de distribuciones de frecuencia simple, tablas de contingencia para la comparación entre grupos, representación gráfica acorde al nivel de medición de las variables y el cálculo de medidas descriptivas en variables numéricas. El contraste de hipótesis para la comparación de grupos se realizó mediante la prueba de asociación de chi cuadrado, bajo un nivel de significancia de 0.05, se utilizó el programa SPSS v.24.

## Resultados

Caracterización de la población:

Los pacientes de cirugía cardiaca enfrentan importantes desafíos en el cuidado de su salud. Según la Tabla 1, las características sociodemográficas determinan que la mayoría de los pacientes son hombres (70.9%), mayores de 60 años (64%), mientras que los cuidadores son mujeres (77.9%) entre 46 y 60 años (50%)

**Tabla 1:** Caracterización sociodemográfica de pacientes de cirugía cardiaca y su cuidador Género principal

Variable	Pacientes n (%)	Cuidadores n (%)
Género (%)		
Masculino	61(70,93)	19(22,09)
Femenino	25(29,07)	67(77,91)
Edad. promedio ± de	57,98 ± 8,43	47,73 ± 12,48
Escolaridad (%)		
Ninguno	10(11,63)	3(3,49)
Primaria	24(27,91)	21(24,42)
Secundaria	32(37,21)	33(38,37)
Técnico	8(9,30)	11(12,79)
Profesional	12(13,95)	18(20,93)
Estado civil (%)		
Soltero(a)	17(19,77)	18(20,93)
Casado(a)	46(53,49)	47(54,65)
Unión libre	17(19,77)	20(23,26)
Viudo(a)	6(6,98)	1(1,16)
Estrato socioeconómico (%)		
Uno	12(13,95)	10(11,63)
Dos	35(40,70)	40(46,51)
Tres	29(33,72)	28(32,56)
Cuatro	5(5,81)	5(5,81)
Cinco	5(5,81)	3(3,49)
Ocupación (%)		
Hogar	34(39,53)	37(43,02)
Empleado	14(16,28)	23(26,74)
Trabajo independiente	18(20,93)	15(17,44)
Estudio	5(5,81)	4(4,65)
Pensionado	15(17,44)	7(8,14)
Tiempo postquirúrgico vs Tiempo de cuidador (%)		
1 - 3 meses	2(2,33)	10(11,63)
4 - 6 meses	25(29,07)	25(29,07)
7 - 9 meses	17(19,77)	11(12,79)
10 - 12 meses	3(3,49)	2(2,33)
Más de un año	39(45,35)	38(44,19)
Parentesco del cuidador (%)		
Esposo(a)		45(52,33)
Hijo(a)		30(34,88)
Hermano(a)		5(5,81)
Sobrino(a)		1(1,16)
Amigo(a)		1(1,16)
Padre / Madre		4(4,65)

**Tabla 2:** Medidas descriptivas para las puntuaciones observadas en la escala ASA.

Dimensión	Ítems	Puntuación máxima posible	Media	Desviación Estándar	Mínimo	Máximo
Interacción social	2	8	5,9	1,1	4	8
Consumo suficiente de alimentos	1	4	3,0	0,8	1	4
Bienestar personal	12	48	36,8	4,9	26	48
Promoción del funcionamiento	4	16	12,1	2,4	7	6
y desarrollo personal						
Actividad y reposo	5	20	13,5	2,5	9	19
Agencia de autocuidado (ASA)	24	96	71,4	9,2	51	90

En la Tabla 2, se encontró un nivel de autocuidado regular frente a interacción social (65.1%), actividad y reposo (60.5%); mientras que la agencia de autocuidado fue buena en el consumo de alimentos (46.5%), en bienestar personal (52.3%), en promoción del funcionamiento y desarrollo personal (47.7%).

En general, el 66.3% de los pacientes presentaron una agencia de autocuidado regular, siendo el 17.4% bajo. Las dimensiones más afectadas son interacción social, actividad y reposo, están relacionadas con las capacidades que facilitan el autocuidado mencionados por Orem en su teoría.

Respecto a la escolaridad del paciente y la capacidad de agencia de autocuidado, se encontró asociación estadísticamente significativa (p = 0,003); a mayor escolaridad mejor empoderamiento del autocuidado. Por ende, la educación brindada a pacientes permite aumentar las capacidades y habilidades para ejecutar acciones de autocuidado^16^.

En el estado civil no se encontraron diferencias (p = 0,461); se observó asociación estadísticamente significativa entre capacidad de agencia de autocuidado y estrato socioeconómico (p < 0,05), así mismo, con la ocupación del paciente (p = 0,008); los recursos económicos limitan algunas acciones de autocuidado^9^, se evidencio que los pensionados tienen mayor empoderamiento.

Se demostró que el autocuidado mejora significativamente con el tiempo postquirúrgico (p = 0,017); el posoperatorio se convierte en escenario de aprendizaje, que a través del tiempo permite mejorar la capacidad de agencia de autocuidado. La Tabla 3 contempla los resultados obtenidos para agencia de autocuidado, según sus características sociodemográficas.

**Tabla 3:** Nivel de agencia de autocuidado del paciente según variables sociodemográficas

Variable	Clasificación Agencia de Autocuidado Baja n (%) Regular n (%) Buena n (%)			Valor p*
Género				0,057
Masculino	14(22,95)	36(59,02)	11(18,03)	
Femenino	1(4,00)	21(84,00)	3(12,00)	
Edad				0,137
35 o menos	0(0,00)	2(100,00)	0(0,00)	
36 - 60	6(20,69)	21(72,41)	2(6,90)	
Mayores de 60	9(16,36)	34(61,82)	12(21,82)	
Escolaridad				0,009
Ninguno	2(20,00)	8(80,00)	0(0,00)	
Primaria	7(29,17)	14(58,33)	3(12,50)	
Secundaria	4(12,50)	26(81,25)	2(6,25)	
Técnico	1(12,50)	4(50,00)	3(37,50)	
Profesional	1(8,33)	5(41,67)	6(50,00)	
Estado civil				0,461
Soltero(a)	3(17,65)	12(70,59)	2(11,76)	
Casado(a)	10(21,74)	26(56,52)	10(21,74)	
Unión libre	1(5,88)	14(82,35)	2(11,76)	
Viudo(a)	1(16,67)	5(83,33)	0(0,00)	
Estrato socioeconómico				0,047
Uno	3(25,00)	9(75,00)	0(0,00)	
Dos	5(14,29)	26(74,29)	4(11,43)	
Tres	6(20,69)	18(62,07)	5(17,24)	
Cuatro	1(20,00)	2(40,00)	2(40,00)	
Cinco	0(0,00)	2(40,00)	3(60,00)	
Ocupación				0,008
Hogar	7(20,59)	25(73,53)	2(5,88)	
Empleado	(7,14)	11(78,57)	2(14,29)	
Trabajo independiente	4(22,22)	12(66,67)	2(11,11)	
Estudio	1(20,00)	4(80,00)	0(0,00)	
Pensionado	2(13,33)	5(33,33)	8(53,33)	
Tiempo postquirúrgico				0,017
1 - 3 meses	0(0,00)	2(100,00)	0(0,00)	
4 - 6 meses	1(4,00)	19(76,00)	5(20,00)	
7 - 9 meses	1(5,88)	10(58,82)	6(35,29)	
10 - 12 meses	0(0,00)	3(100,00)	0(0,00)	
Más de un año	13(33,33)	23(58,97)	3(7,69)	
Nivel de sobrecarga				0,000
No sobrecarga	6(25%)	49(71,01%)	14(20,29%)	
Leve	4(36,36%)	7(63,64%)		
Intensa	5(83,33%)	1(16,67%)		

*prueba de Chi cuadrado de independencia, Nivel de significancia establecido: 0.05

Respecto al nivel de sobrecarga de los cuidadores, en la Tabla 4 se detallan las medidas descriptivas para puntuaciones observadas en escala Zarit; en general, se observó un promedio por cuidador de 30.2 ± 16.8 puntos, con rango entre 4 y 63 puntos. Acorde con las puntuaciones observadas, el 80.2% de cuidadores no presentó sobrecarga y el 7% un nivel de sobrecarga intenso respecto a su actividad como cuidador. Sin embargo, se evidencio que las mujeres cuidadoras expresan mayor nivel de sobrecarga, sin diferencias significativas respecto al grupo de hombres cuidadores (p = 0,175). Las personas dedicadas a labores del hogar expresan mayor nivel de sobrecarga, también presente en personas con trabajo independiente.

Cuidadores de 35 años o menos expresan mayor nivel de sobrecarga, aunque sin diferencias significativas respecto a cuidadores entre 36 yVV 60 años, y mayores de 60 años (p = 0,090). Por otra parte, existe asociación, aunque no significativa entre escolaridad del cuidador y nivel de sobrecarga (p = 0,223); cuidadores sin nivel educativo expresan mayor proporción de sobrecarga intensa (66.7%), asociado al déficit de conocimientos sobre el cuidado. Aunque, la prevalencia de sobrecarga intensa es mayor en personas con pareja estable, no se evidenciaron diferencias significativas respecto al estado civil (p = 0,974).

El nivel de sobrecarga intensa predomina en cuidadores entre 1 y 3 meses realizando la labor (10%) o quienes superan el año siendo cuidadores (13.2%). A mayor tiempo de exposición al cuidado de su familiar, existe mayor nivel de sobrecarga, aunque no se puede concluir que exista asociación estadísticamente significativa (p = 0,063). Al afrontar el rol de cuidado inicialmente se enfrentan a situaciones desconocidas para ellos, lo que conlleva a problemas de salud, laborales, económicos y sociales^17^.

**Tabla 4:** Nivel de sobrecarga del cuidador según variables sociodemográficas

	Clasificación del nivel de sobrecarga (Zarit)			
Variable	No sobrecarga	Sobrecarga leve	Sobrecarga intense del nivel de sobrecarga (Zarit)	Valor p
	n (%)	n (%)	n (%)	
Género del cuidador		5(26,3)	1(5,3)	0,175
Masculino	13(68,4)			
Femenino	56(83,6)	6(9,0)	5(7,5)	
Edad del cuidador	11(61,1)	5(27,8)	2(11,1)	0,090
35 o menos				
36-60	44(83,0)	6(11,3)	3(5,7)	
Mayores de 60	14(93,3)	0(0,0)	1(6,7)	
Escolaridad del cuidador		0(0,0)	2(66,7)	0,223
Ninguno	1(33,3)			
Primaria	16(76,2)	4(19,0)	1(4,8)	
Secundaria	28(84,8)	3(9,1)	2(6,1)	
Técnico	10(90,9)	1(9,1)	0(0,0)	
Profesional	14(77,8)	3(16,7)	1(5,6)	
Estado civil del cuidador				0,974
Soltero(a)	15(83,3)	2(11,1)	1(5,6)	
Casado(a)	36(76,6)	7(14,9)	4(8,5)	
Unión libre	17(85,0)	2(10,0)	1(5,0)	
Viudo(a)	1(100,0)	0(0,0)	0(0,0)	
Estrato socioeconómico del cuidador		0(0,0)	1(10,0)	
Uno	9(90,0)			0,608
Dos	30(75,0)	6(15,0)	4(10,0)	
Tres	23(82,1)	4(14,3)	1(3,6)	
Cuatro	4(80,0)	1(20,0)	0(0,0)	
Cinco	3(100,0)	0(0,0)	0(0,0)	
Ocupación del cuidador				0,328
Hogar	29(78,4)	4(10,8)	4(10,8)	
Empleado	19(82,6)	4(17,4)	0(0,0)	
Trabajo independiente	11(73,3)	2(13,3)	2(13,3)	
Estudio	3(75,0)	1(25,0)	0(0,0)	
Pensionado	7(100,0)	0(0,0)	0(0,0)	
Tiempo de cuidador				0,063
1 - 3 meses	6(60,0)	3(30,0)	1(10,0)	
4 - 6 meses	23(92,0)	2(8,0)	0(0,0)	
7 - 9 meses	11(100,0)	0(0,0)	0(0,0)	
10 - 12 meses	2(100,0)	0(0,0)	0(0,0)	
Más de un año	27(71,1)	6(15,8)	5(13,2)	

Clasificación del nivel de sobrecarga (Zarit)

Correlación entre la agencia de autocuidado y nivel de sobrecarga del cuidador:

Cuidadores como esposo(a) o hijos(as), tienen mayor nivel de sobrecarga, este rol es desempeñado en la mayoría de los casos por familiares en primer grado; no se evidenciaron diferencias significativas frente al parentesco con el paciente (p = 0,463). Esta investigación, demuestra que existe una asociación estadísticamente significativa entre el nivel de sobrecarga de los cuidadores y la capacidad de agencia de autocuidado de los pacientes (p < 0,05), a mayor sobrecarga del cuidador menor capacidad de agencia de autocuidado en los pacientes. En cuidadores con una sobrecarga intensa, el 83,33% tenían a su cargo pacientes con una baja capacidad de autocuidado. En el grupo de cuidadores con sobrecarga leve, el 63% tenía a su cargo pacientes con una capacidad de agencia de autocuidado media, mientras que, en el grupo de cuidadores sin sobrecarga, la mayoría de sus pacientes (71, 01%) tenía una capacidad de agencia de autocuidado media, pero un 20,29% exhibió una alta capacidad de autocuidado.

## Discusión

En esta investigación el cuidador principal es la mujer, superando el 70% como en Portugal, México, España, Chile; las personas cuidadas son adultos mayores, tienen unión libre, contrario a otros estudios, donde predomina el estar casado en más de la mitad de cuidadores de Portugal, México, y Cuba^18^. Respecto a la ocupación, prevalecen las labores del hogar. En Colombia la Encuesta Nacional Uso del Tiempo 2012-2013^26^, evidencia que el 22,8% dedica su tiempo al cuidado de familiares^19^. Así mismo, se evidencia que la labor de cuidador está relacionada con el tiempo de posoperatorio.

Existen similitudes con otros estudios^18^^-^^19^^-^^20^^-^^21^, donde la interacción social, actividad y reposo estaban en nivel regular, mientras que bienestar personal, promoción del funcionamiento y desarrollo personal mostraron niveles buenos de autocuidado. Se considera que el ejercicio regular y la adherencia farmacológica se asocian con un mejor funcionamiento fisiológico^22^. El rol de cuidador es desempeñado por esposos o hijos, quienes tienen mayor nivel educativo que los pacientes, y se encuentran casados o en unión libre; se evidenció que los pacientes no tienen una relación marital, y predomina la ocupación el hogar. Sumado a ello, la escolaridad del paciente es baja, siendo la población casada la más representativa, varios de ellos no tienen una vida laboral activa por lo cual se dedican a labores del hogar. Sin embargo, el cuidador principal son mujeres en promedio 60 años, presentan depresión asociada con la percepción de carencias económicas básicas para el cuidado^23^.

Otros estudios^16^^-^^24^, encontraron que la mayoría de los participantes reportaron un rango alto de agencia de autocuidado, siendo las mujeres con mayor agencia de autocuidado. También se evidencia que la agencia de autocuidado mejora con la edad; siendo las personas jóvenes con menor participación en actividades de autocuidado debido a la competencia social y educativa, a los compromisos familiares y laborales. En cuanto a la ocupación y el nivel de sobrecarga, las mujeres tienen mayor nivel de sobrecarga, derivado del acto de cuidar^25^. Se señala que existe relación entre la carga percibida por cuidadores y el nivel de dependencia del paciente^26^^-^^27^^-^^28^.

Esta investigación demuestra, que la capacidad de agencia de autocuidado adecuada reduce el nivel de sobrecarga del cuidador, cuando el paciente logra adaptarse a las actividades que contribuyen al cuidado, disminuye el nivel de dependencia, generando menor carga al cuidador principal permitiendo un estilo de vida compatible entre el rol de cuidar y el desarrollo personal.

Enfermería debe incentivar y motivar las intervenciones multidisciplinarias para fortalecer el cuidado de la salud de los individuos y su entorno, comprendiendo los fenómenos que existen en la práctica del cuidado.

Las limitaciones del estudio fue la recolección de la muestra establecida por la falta de contacto del paciente, mortalidad y deseo de no participación. Se sugiere continuar realizando estudios como este, en particular realizar réplicas de este estudio considerando otras variables como el nivel de ansiedad de pacientes y cuidadores.

## Conclusión

Cuidar trae consigo diferentes repercusiones, que afectan la salud física, psicológica y emocional del cuidador generando sobrecarga; de tal modo, al determinar la relación existente entre el nivel de sobrecarga del cuidador y la agencia de autocuidado del paciente de cirugía cardiaca, se demuestra que existe una asociación inversa y estadísticamente significativa, a mayor capacidad de agencia de autocuidado, menor nivel de sobrecarga del cuidador. Sin embargo, el nivel de agencia de autocuidado de los pacientes y el nivel de sobrecarga del cuidador depende de sus características sociodemográficas como la edad, el sexo, el estado civil, el nivel socioeconómico y la ocupación. Cabe resaltar, que el nivel de agencia de autocuidado mejora con el tiempo de recuperación y el nivel de sobrecarga del cuidador está relacionado con el tiempo que lleva desempeñando su rol.

Enfermería debe identificar el nivel de agencia de autocuidado del paciente de cirugía cardiaca durante su posoperatorio para intervenir oportunamente desde la práctica del cuidado dando respuesta a las necesidades que experimenta el paciente y su cuidador principal.
